# Association of Field Position and Career Length With Risk of Neurodegenerative Disease in Male Former Professional Soccer Players

**DOI:** 10.1001/jamaneurol.2021.2403

**Published:** 2021-08-02

**Authors:** Emma R. Russell, Daniel F. Mackay, Katy Stewart, John A. MacLean, Jill P. Pell, William Stewart

**Affiliations:** 1 Institute of Neuroscience and Psychology, University of Glasgow, Glasgow, United Kingdom; 2 Institute of Health and Wellbeing, University of Glasgow, Glasgow, United Kingdom; 3 Institute of Cardiovascular and Medical Sciences, University of Glasgow, Glasgow, United Kingdom; 4 Hampden Sports Clinic, Hampden Stadium, Glasgow, United Kingdom; 5 Department of Neuropathology, Queen Elizabeth University Hospital, Glasgow, United Kingdom

## Abstract

**Importance:**

Neurodegenerative disease mortality is higher among former professional soccer players than general population controls. However, the factors contributing to increased neurodegenerative disease mortality in this population remain uncertain.

**Objective:**

To investigate the association of field position, professional career length, and playing era with risk of neurodegenerative disease among male former professional soccer players.

**Design, Setting, and Participants:**

This cohort study used population-based health record linkage in Scotland to evaluate risk among 7676 male former professional soccer players born between January 1, 1900, and January 1, 1977, and 23 028 general population control individuals matched by year of birth, sex, and area socioeconomic status providing 1 812 722 person-years of follow-up. Scottish Morbidity Record and death certification data were available from January 1, 1981, to December 31, 2016, and prescribing data were available from January 1, 2009, to December 31, 2016. Database interrogation was performed on December 10, 2018, and data were analyzed between April 2020 and May 2021.

**Exposures:**

Participation in men’s soccer at a professional level.

**Main Outcomes and Measures:**

Outcomes were obtained by individual-level record linkage to national electronic records of mental health and general hospital inpatient and day-case admissions as well as prescribing information and death certification. Risk of neurodegenerative disease was evaluated between former professional soccer players and matched general population control individuals.

**Results:**

In this cohort study of 30 704 male individuals, 386 of 7676 former soccer players (5.0%) and 366 of 23 028 matched population control individuals (1.6%) were identified with a neurodegenerative disease diagnosis (hazard ratio [HR], 3.66; 95% CI, 2.88-4.65; *P* < .001). Compared with the risk among general population control individuals, risk of neurodegenerative disease was highest for defenders (HR, 4.98; 95% CI, 3.18-7.79; *P* < .001) and lowest for goalkeepers (HR, 1.83; 95% CI, 0.93-3.60; *P* = .08). Regarding career length, risk was highest among former soccer players with professional career lengths longer than 15 years (HR, 5.20; 95% CI, 3.17-8.51; *P* < .001). Regarding playing era, risk remained similar for all players born between 1910 and 1969.

**Conclusions and Relevance:**

The differences in risk of neurodegenerative disease observed in this cohort study imply increased risk with exposure to factors more often associated with nongoalkeeper positions, with no evidence this association has changed over the era studied. While investigations to confirm specific factors contributing to increased risk of neurodegenerative disease among professional soccer players are required, strategies directed toward reducing head impact exposure may be advisable in the meantime.

## Key Points

**Question:**

What is the association of field position, career length, and playing era with risk of neurodegenerative disease in male former professional soccer players?

**Findings:**

In this cohort study of 7676 Scottish male former professional soccer players and 23 028 general population control individuals matched by sex, year of birth, and area socioeconomic status, risk of neurodegenerative disease among former soccer players varied by field position and career length but was similar across era of participation.

**Meaning:**

In this study, risk of neurodegenerative disease was higher among former professional soccer players with longer careers and among those in nongoalkeeper positions, indicating that factors associated with nongoalkeeper positions should be scrutinized to mitigate risk; meanwhile, strategies to reduce head impact exposure may be advisable to reduce negative outcomes in this population.

## Teaser

This cohort study investigates the association of field position, professional career length, and playing era with risk of neurodegenerative disease among Scottish male former professional soccer players.

## Introduction

Internationally, an estimated 50 million individuals are living with dementia, resulting in an approximate annual cost to the global economy of $1 trillion.^1^ As such, studies to identify potential modifiable factors associated with risk of dementia and other neurodegenerative diseases are essential in providing opportunities for interventions that might reduce disease burden.^2^ Former professional contact sports athletes are recognized as having higher mortality from dementia^3,4^ and a range of other neurodegenerative diseases, including motor neuron disease^3,4,5,6,7,8^ and Parkinson disease,^3,4^ compared with the general population. Autopsy studies in these populations frequently report the presence of a specific neurodegenerative pathology associated with traumatic brain injury (TBI), known as chronic traumatic encephalopathy (CTE) neuropathologic change.^9^ Nevertheless, it remains uncertain whether risk of neurodegenerative disease in former athletes is associated with TBI and repetitive head impact exposure or other factors as yet unidentified.

The association between TBI in sports and late neurodegenerative disease was first recognized in the early 20th century in descriptions of the so-called punch-drunk syndrome of former boxers,^10^ with more recent recognition of CTE neuropathologic change in autopsy studies of nonboxer athletes and others exposed to TBI bringing this issue to wider attention.^11,12^ To date, virtually all individuals in whom CTE neuropathologic change is described have prior history of TBI or repetitive head impact exposure.^9,10,11,12,13,14^ Although CTE neuropathologic change is a frequent pathology in contact sports athletes with dementia, neurodegenerative pathologies and diagnoses in these populations are not restricted to CTE.^12^

Outside autopsy studies on convenience samples of research brain donations, population-level data provide evidence of increased neurodegenerative risk among former athletes. Specifically, neurodegenerative mortality was reported at higher-than-expected rates among former professional US football players in the National Football League^3,15^ and former professional soccer players.^4,5,6,7,8^ Mirroring experience from autopsy observations, these studies document higher neurodegenerative disease risk across a range of diagnoses. Thus, while overall neurodegenerative disease mortality was approximately 3.5-fold higher in former professional soccer players than in matched general population control individuals, risk varied from a 2-fold increase in deaths with Parkinson disease to a 5-fold increase in deaths with Alzheimer disease.^4^

Played in more than 200 countries and with more than one-quarter of a billion active participants, soccer is the world’s most popular participation sport.^16^ Although data suggest TBIs are infrequent in soccer,^17^ training and gameplay routinely involve exposure to repetitive head impacts through soccer ball heading. Furthermore, while data are limited, evidence suggests risk of TBI and participation in heading are in part dependent on field position, with risk of head injury in goalkeepers around one-third that of players in other positions^17^ and heading by goalkeepers being exceptionally rare.^18^ In this context, we hypothesized that risk of neurodegenerative disease among former professional soccer players might vary by field position and career length, with higher risk associated with outfield (ie, nongoalkeeper) positions and increasing career length. Further, reflecting changes in game technology and medical care in the period under study, we hypothesized that risk of neurodegenerative disease might vary by playing era. To address these hypotheses, we accessed national electronic health records to explore the association of field position, professional career length, and playing era with risk of neurodegenerative disease among Scottish male former professional soccer players.

## Methods

### Approvals

Ethical approval was provided by the University of Glasgow College of Medical, Veterinary and Life Sciences Ethics Committee (project number 200160147), with protocol and data governance procedures reviewed and approved by National Health Service Scotland’s Public Benefit and Privacy Panel for Health and Social Care (reference 1718-0120). As all health record data were anonymized to researchers, participant consent was not required. The complete protocol for the Football’s Influence on Lifelong Health and Dementia Risk (FIELD) study is published elsewhere.^19^ The analysis and reporting of this study are consistent with the Strengthening the Reporting of Observational Studies in Epidemiology (STROBE) reporting guideline.^20^

### Cohort Identification and Inclusion Criteria

Former professional soccer players were identified through the Record of Pre-War Scottish League Players version 2^21^ and the Record of Post-War Scottish League Players version 6^22^ compiled from archives of the Scottish Football Museum and individual league clubs. Available data included player demographic characteristics (full name and date of birth) and career information (dates of first signing and retirement and player position). The databases were merged and duplicates deleted. Study inclusion was restricted to individuals 40 years or older as of December 31, 2016. Individuals with missing or incomplete date of birth were excluded. All individuals included in this study were male, as identified by electronic health records classification, reflecting the male professional soccer league structure over the period of study. Full details on the professional soccer players and individuals in the matched control cohort are published elsewhere.^4^ The present analysis of all data sets for outcomes, field position, career length, and playing era was prespecified in the published protocol.^19^

### Matched Population Control Individuals

Probabilistic matching was applied to the full name and date of birth of former soccer players to link them to their unique community health index. The community health index database was used to randomly identify population control individuals as male and match them with the former soccer players on a 3:1 ratio by year of birth and quintile of postcode level area socioeconomic status. The National Health Service Information Services Division records last-known postcode of residence for all individuals from which area socioeconomic deprivation is calculated using the Scottish Index of Multiple Deprivation, which is derived from information on income, employment, health, education, housing, and crime.^23^ The Scottish Index of Multiple Deprivation is categorized into quintiles ranging from 1 (most deprived) to 5 (most affluent).

### Field Position, Career Length, and Era of Play

Former soccer players were categorized according to their principal field position as either goalkeeper or outfield player, and the latter further subdivided as defender, midfielder, forward or, where a player had participated in several field positions, multiposition. Professional career length was defined as the time between the date of first signing to a professional soccer club and date of retirement from professional soccer and did not include years playing nonprofessional soccer or participation at the managerial level. Professional career length was treated as an ordinal variable and categorized as short (less than 5 years), low medium (6 to 10 years), high medium (11 to 15 years), or long (more than 15 years). Outliers, defined as those with a career length greater than 2 standard deviations above the mean, were excluded from analyses. The association between era of professional soccer career and neurodegenerative disease outcome was examined by analyzing year of birth data in groups of 2 decades spanning 1910 to 1969. Neurodegenerative disease diagnoses in players and control individuals born prior to 1910 and after 1969 were too few to permit meaningful analysis.

### Neurodegenerative Disease Diagnoses

Outcomes for all individuals, whether living or deceased at time of data capture, were obtained by individual-level record linkage to hospitalizations held within Scottish Morbidity Record SMR01 (General/Acute Inpatient and Day Case) and Scottish Morbidity Record SMR04 (Mental Health Inpatient and Day Case) data sets, dispensed prescriptions held within the Scottish National Prescribing Information System, and death certification. SMR01, SMR04 and death certification data sets are coded using the *International Classification of Diseases, Ninth Revision* (*ICD-9*) and *ICD-10*. The *ICD-9* and *ICD-10* codes used to ascertain neurodegenerative disease identified all neurodegenerative diseases, including dementia not otherwise specified, Alzheimer disease, non-Alzheimer dementia, motor neuron disease, and Parkinson disease (Table 1). The Scottish National Prescribing Information System records every prescription dispensed in the community, with medications coded using the British National Formulary.^24^ For the purposes of this study, prescriptions including medication coded under Section 4.11, Drugs for Dementia, were captured. SMR01, SMR04, and death certification data were available from January 1, 1981, to December 31, 2016, and prescribing data were available from January 1, 2009, to December 31, 2016. Database interrogation was performed on December 10, 2018, and data were analyzed between April 2020 and May 2021.

**Table 1.** *International Classification of Diseases, Ninth Revision* (*ICD-9*) and *ICD-10* Codes Used to Ascertain Neurodegenerative Disease

Disease	*ICD-9*	*ICD-10*
All neurodegenerative disease	290.0-290.4; 294.1; 294.2; 331.0-331.2; 331.6-331.9; 332; 335.2	F01-F03; G12.2; G20; G21; G23.1-G23.3; G23.8; G23.9; G30; G31
Dementia not otherwise specified	290.0-290.4; 294.1, 294.2; 331.0; 331.1, 331.2; 331.6-331.9	F01-F03; G23.1-G23.3; G30; G31
Alzheimer disease	331.0	G30
Non-Alzheimer dementia	290.0-290.4; 294.1 294.2; 331.1, 331.2; 331.6-331.9	F01-F03; G23.1- G23.3; G31
Motor neuron disease (amyotrophic lateral sclerosis)	335.2	G12.2
Parkinson disease	332	G20; G21; G23.8; G23.9

### Statistical Analysis

Incident neurodegenerative disease diagnoses for former players and matched controls were ascertained from the earliest coding of neurodegenerative disease captured from either hospitalization, death certification, or prescribing data sets. Cox proportional hazards regression was run for each group analysis to assess differences in neurodegenerative disease outcomes between former soccer players and their matched general population controls, with results reported as hazard ratios (HR) and 95% CIs. In separate sensitivity analyses, neurodegenerative disease outcomes were subjected to competing risks regression analyses to establish whether the estimated HRs were attenuated by the competing risk of death from causes other than neurodegenerative disease.^25^ Standard logistic regression was used to compare risk of neurodegenerative disease among subgroups of former players defined by player position, with adjustment for confounders of age and socioeconomic status. A likelihood ratio test was performed to assess the associations between year of birth and career length with neurodegenerative disease outcomes. All statistical analyses were undertaken using Stata version 16 (StataCorp), with statistical significance set at 2-sided *P* < .05.

## Results

### Player Field Position and Neurodegenerative Disease

**Figure 1.**
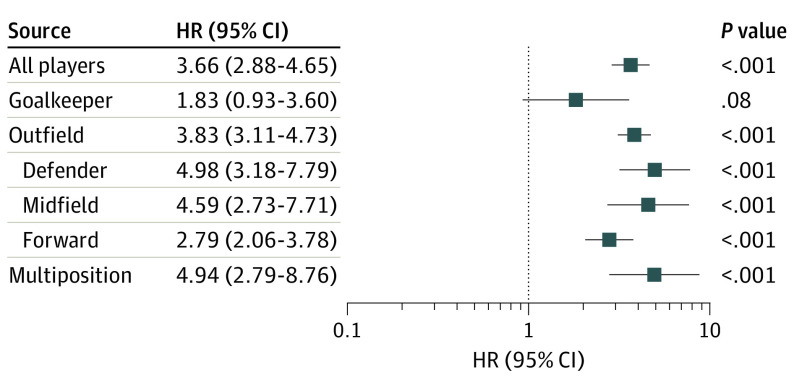
Player Position and Risk of Neurodegenerative Disease Among Male Former Professional Soccer Players Compared With a Matched General Population Control Group HR indicates hazard ratio.

Over a median (interquartile range) follow-up of 18 years (10.0-27.4), 386 of 7676 former professional soccer players (5.0%) and 366 of 23 028 matched population control individuals (1.6%) were identified with neurodegenerative disease diagnoses (HR, 3.66; 95% CI, 2.88-4.65; *P* < .001) (Table 2). Field position data were available for 6622 former soccer players. Compared with matched population control individuals, risk of neurodegenerative disease varied by field position. Specifically, although neurodegenerative disease risk was higher in goalkeepers than in matched control individuals, this difference was not statistically significant (HR, 1.83; 95% CI, 0.93-3.60; *P* = .08) and was attenuated after adjustment for the competing risk of deaths from nonneurodegenerative disease (eTable 1 in the Supplement). In contrast, outfield player positions showed higher risk of neurodegenerative disease compared with matched control individuals (HR, 3.83; 95% CI, 3.11-4.73; *P* < .001), which was not attenuated after adjustment for deaths from nonneurodegenerative disease (eTable 1 in the Supplement) and was higher than risk among goalkeepers (odds ratio, 2.22; 95% CI, 1.35-3.64; *P* = .002). Risk of neurodegenerative disease varied among outfield player positions and was lowest for forwards (HR, 2.79; 95% CI, 2.06-3.78; *P* < .001) and highest for defenders (HR, 4.98; 95% CI, 3.18-7.79; *P* < .001; odds ratio for defenders vs forwards, 1.52; 95% CI, 1.12-2.07; *P* = .008) (Figure 1; eTable 1 in the Supplement).

**Table 2.** Neurodegenerative Disease Diagnoses by Data Source and Subtype

Disease	No. (%)		Hazard ratio (95% CI)	*P* value^a^
	Former soccer players (n = 7676)	Matched controls (n = 23 028)		
All neurodegenerative disease diagnoses	386 (5.0)	366 (1.6)	3.66 (2.88-4.65)	<.001
Death certification	222 (2.9)	228 (1.0)	3.53 (2.72-4.57)	<.001
Hospitalization	149 (1.9)	141 (0.6)	3.10 (2.31-4.18)	<.001
Drug prescription for dementia	199 (2.6)	144 (0.6)	4.51 (3.38-6.01)	<.001
Dementia not otherwise specified^b^	338 (4.4)	312 (1.4)	3.59 (2.93-4.39)	<.001
Motor neuron disease^c^	24 (0.3)	23 (0.1)	3.52 (1.81-6.88)	<.001
Parkinson disease^c^	28 (0.4)	45 (0.2)	2.09 (1.20-3.61)	.009

^a^ Cox proportional hazards regression.

^b^ Data derived from death certification, hospitalization, and prescribing information.

^c^ Data derived from death certification and hospitalization records.

### Career Length and Neurodegenerative Disease

**Figure 2.**
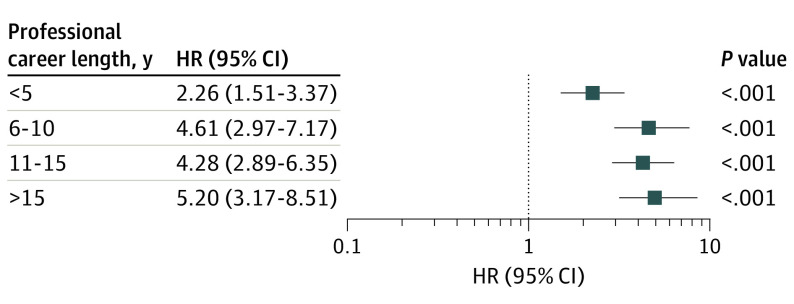
Career Length and Risk of Neurodegenerative Disease Among Male Former Professional Soccer Players Compared With a Matched General Population Control Group HR indicates hazard ratio.

The mean (SD) career length for professional outfield soccer players was 8.6 (6.2) years, typically spanning age 20.0 (2.7) years to retirement at age 28.5 (5.6) years. Considering career length in 5-year bands revealed that risk of neurodegenerative disease increased with increasing career length (likelihood ratio χ^2^_3_, 10.75; *P* = .01). Specifically, those with short career length showed lowest risk of neurodegenerative disease compared with matched control individuals (HR, 2.26; 95% CI, 1.51-3.37; *P* < .001), while risk was greatest in those with the longest careers (HR, 5.20; 95% CI, 3.17-8.51; *P* < .001) (Figure 2; eTable 2 in the Supplement).

### Era of Play and Neurodegenerative Disease

**Figure 3.**
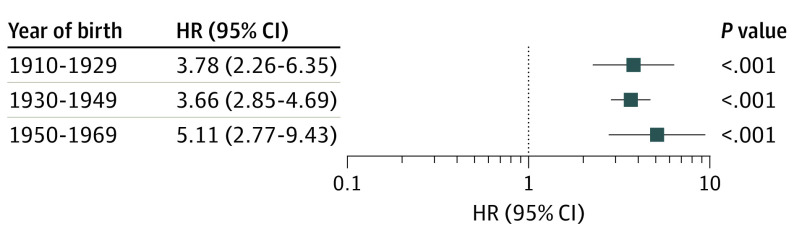
Date of Birth and Risk of Neurodegenerative Disease Among Male Former Professional Soccer Players Compared With a Matched General Population Control Group HR indicates hazard ratio.

Risk of neurodegenerative disease among former professional soccer players was considered in association with playing era, with soccer players divided into 2-decade subgroups by year of birth. Compared with matched general population controls, risk of neurodegenerative disease diagnosis remained similar across all birth cohorts for former professional outfield soccer players born from 1910 to 1969 (χ^2^_2_, 0.46; *P* = .79) (Figure 3; eTable 3 in the Supplement).

## Discussion

In this retrospective cohort study, risk of neurodegenerative disease among a cohort of male former professional soccer players was associated with field position and career length. While overall risk of neurodegenerative disease was around 3.5 times higher in former professional soccer players than in the matched general population control individuals, we found no significant increase in risk of neurodegenerative disease among goalkeepers. In contrast, risk was high across all outfield positions and highest in defenders, who had an approximately 5-fold increase in risk of neurodegenerative disease compared with the matched general population control individuals. Further, risk of neurodegenerative disease among outfield players increased with career length. In contrast, risk of neurodegenerative disease remained similar for former soccer players born between 1910 and 1969.

We previously reported neurodegenerative disease mortality among former professional soccer players was approximately 3.5-fold higher than among matched general population controls.^4^ However, death certification records are subject to error, with neurodegenerative disease diagnoses often underreported.^26^ To address this, the present analysis included all available health records to identify incident neurodegenerative disease in both community and hospital settings, as well as deaths. Using this more complete ascertainment of neurodegenerative disease, we confirmed an approximately 3.5-fold higher risk of neurodegenerative disease among a cohort of Scottish male former professional soccer players, which corroborates our previous mortality analysis.^4^

The observation of CTE neuropathologic change in autopsy studies of former contact sports athletes,^11,12,13,14^ including former soccer players,^12^ is suggested as evidence that neurodegenerative disease in these populations might reflect late outcomes from TBI or repetitive subconcussive head impacts.^11^ We have no access to direct information on head impact exposure or TBI in our study populations. Nevertheless, purposeful head impacts through heading the ball are part of gameplay and training in soccer, with research demonstrating evidence of immediate, short-lived cognitive impairment^27^ and brain structural changes on imaging following soccer ball heading.^28^ Further, TBI risk^17^ and heading^18^ exposure varies by field position, being lower among goalkeepers than players in other field positions. As such, we hypothesized goalkeepers as a population at low risk and outfield players at high risk. While goalkeepers showed slightly higher risk of neurodegenerative disease than the matched control population, this observation did not reach our prespecified cutoff for statistical significance, possibly reflecting insufficient numbers in our data set to power the analysis. Outfield player positions, by contrast, showed significantly higher neurodegenerative disease compared with both goalkeepers and the matched general population control individuals. Follow-on studies including larger numbers are required to further explore risk of neurodegenerative disease among specific field positions.

The data demonstrate an association between soccer career length and risk of neurodegenerative disease, with highest risk among those with the longest playing careers. A similar association between career length and risk of autopsy-confirmed CTE neuropathologic change has been noted among former American football athletes.^29^ These observations are consistent with the cumulative effect of exposure to a risk factor within the sport. Taken together with our observation that outfield players were at higher risk of neurodegenerative disease than goalkeepers, these data provide further evidence in support of exposure to TBI and repetitive head impacts as factors associated with risk of neurodegenerative disease.

The cohort in this study included Scottish professional soccer players born between January 1, 1900, and December 31, 1976. With typical careers spanning from age 20 years to just younger than 30 years, many of the players in this cohort were participating in professional soccer into the first decade of the 21st century. There have been many changes in soccer over the study period that might affect risk of neurodegenerative disease. Although the regulation dry weight of an association soccer ball has not changed since 1872, throughout much of the last century, soccer balls were composed of an inner air sac, typically rubber, covered with an outer leather shell.^30^ Toward the latter half of the 20th century, this leather covering was replaced by a synthetic shell, with the benefit that the synthetic covering does not absorb water.^31^ Our data demonstrate that risk of neurodegenerative disease among former professional soccer players remained similar across players born in an era when solely leather balls would be used to players born in an era when there was a transition from leather to synthetic balls.^30^ The current data set does not permit analysis of outcomes in players participating in an era when the soccer ball was solely synthetic. Further, there have been advances in sports medicine in recent decades, particularly around the assessment and management of symptomatic head injuries.^11^ As such, whether risk remains high in soccer players born after 1969 remains unknown.

### Strengths and Limitations

A strength of this work is inclusion of a comprehensive cohort of male former professional soccer players, providing what is, to our best knowledge, the largest such study to date in a single sport. Further, we assessed outcomes in former soccer players against general population control individuals matched by age, sex, and socioeconomic status. The Scottish Index of Multiple Deprivation considers among its assessment criteria access to health care and income in each postcode area.^23^ By matching for area socioeconomic status, potential biases attributable to access to the National Health Service and limited private health care in Scotland were controlled for. We have no reason to believe there would be any systematic difference in health care access or in accuracy of recording of hospital admissions, prescribing information, or death certification between the former soccer players and control individuals in this study.

This study had limitations. An important limitation is an absence of wider information on soccer participation among the professional soccer players in this study, including age at first participation, duration of any nonprofessional participation in soccer, and participation in other contact sports. Furthermore, given soccer’s popularity, some of the population control individuals are likely to have participated in soccer below the professional level, a factor that may be associated with risk of neurodegenerative disease. Nevertheless, increased risk of neurodegenerative disease remained evident among the former professional soccer players in this study when compared with matched population control individuals, consistent with risk from professional soccer participation being over and above any population risk from amateur-level participation. Given the potential public health impact, future clinical research and population surveillance initiatives in dementia should capture information on previous contact sport participation to permit analysis of the contribution of nonprofessional exposures to dementia risk.

While we recognize electronic health records have many strengths, they also present limitations. For example, we could not access information on wider known factors associated with risk of neurodegenerative disease. However, as indirect insights into these factors, we have reported lower cardiovascular deaths^4^ and lower hospitalization rates for common mental health disorders,^32^ including admissions for drug and alcohol use disorders, among former soccer players. Future studies might consider the association of such factors with neurodegenerative disease outcomes in former contact sports athletes.

## Conclusions

In this retrospective cohort study, risk of neurodegenerative disease was higher among male former professional soccer players than among general population control individuals matched by age, sex, and area socioeconomic status. Further, risk of neurodegenerative disease in former soccer players was associated with field position and with longer career lengths, implying higher risk with cumulative exposure to factors more often associated with outfield positions. There is a need for further studies to interrogate the association between soccer and neurodegenerative disease, including risks in amateur and youth soccer. Meanwhile, adopting a precautionary principle approach to mitigate risk of neurodegenerative disease by reducing exposure to TBI and head impacts in soccer and wider sports might be advised.

## Notes

Supplement. **eTable 1.** Player position and risk of neurodegenerative disease **eTable 2.** Career length and risk of neurodegenerative disease **eTable 3.** Date of birth and risk of neurodegenerative disease
