# Supplementary material for: Association of Field Position and Career Length With Risk of Neurodegenerative Disease in Male Former Professional Soccer Players
Source: JAMA Neurol. 2021 Aug 2;78(9):1–7. doi: 10.1001/jamaneurol.2021.2403 (PMC8329793; doi:10.1001/jamaneurol.2021.2403)
Supplement: Supplement. — eTable 1. Player position and risk of neurodegenerative disease eTable 2. Career length and risk of neurodegenerative disease eTable 3. Date of birth and risk of neurodegenerative disease [file jamaneurol-e212403-s001.pdf]

Supplemental Online Content

Russell ER, Mackay DF, Stewart K, MacLean JA, Pell JP, Stewart W. Association of field position and career length with risk of neurodegenerative disease in male former professional soccer players. *JAMA Neurol*. Published online August 2, 2021. doi:10.1001/jamaneurol.2021.2403

eTable 1. Player position and risk of neurodegenerative disease

eTable 2. Career length and risk of neurodegenerative disease

eTable 3. Date of birth and risk of neurodegenerative disease

This supplemental material has been provided by the authors to give readers additional information about their work.

© 2021 Russell ER et al. *JAMA Neurology.*

eTable 1: Player position and risk of neurodegenerative disease

	Former Soccer Players	Matched Population Controls	Hazard Ratio (95% CI)	*P**	Subhazard ratio (95% CI)	*P***
	**n/total n (%)**	**n/total n (%)**				
**All players**	386/7,676 (5·0)	366/23,028 1·6)	3·66 (2·88, 4·65)	<0·001	3·90 (3·25, 4·66)	<0·001
**Goalkeeper**	19/598 (3·2)	34/1,794 (1·9)	1·83 (0·93, 3·60)	0·079	1·09 (0·49, 2·44)	0·883
**Outfield**	271/4,813 (5·6)	234/14,439 (1·6)	3·83 (3·11, 4·73)	<0.001	4·48 (3·67, 5·48)	<0·001
Defender	81/1,347 (6·0)	55/4,041 (1·4)	4·98 (3·18, 7·79)	<0·001	5·56 (3·63, 8·49)	<0·001
Midfield	57/1,129 (5·0)	40/3,387 (1·2)	4·59 (2·73, 7·71)	<0·001	6·04 (3·63, 10·05)	<0·001
Forward	133/2,337(5·7)	139/7,011 (2·0)	2·79 (2·06, 3·78)	<0·001	3·37 (2·51, 4·51)	<0·001
**Multiposition**	45/1,211 (3·7)	30/3,633 (0·8)	4·94 (2·79, 8·76)	<0·001	5·27 (2·99, 9·30)	<0·001

*Cox proportional hazards regression; **Adjusted for the competing risk of deaths from causes other than neurodegenerative disease.

© 2021 Russell ER et al. *JAMA Neurology.*

eTable 2: Career length and risk of neurodegenerative disease

	**Former Soccer Players**	**Matched Population Controls**	**Hazard Ratio (95% CI)**	***P****	**Subhazard ratio (95% CI)**	***P*****
	**n/total n (%)**	**n/total n (%)**				
**1-5 years**	70/2,037 (3.4)	81/6,111 (1.3)	2·26 (1·51, 3·37)	<0·001	2·53 (1·74, 3·67)	<0·001
**6-10 years**	73/1,535 (4.8)	62/4,605 (1.3)	4·61 (2·97, 7·17)	<0·001	4·75 (3·07, 7·37)	<0·001
**11-15 years**	96/1,354 (7.1)	82/4,062 (2.0)	4·28 (2·89, 6·35)	<0·001	5·09 (3·52, 7·37)	<0·001
**>15 years**	72/978 (7.4)	36/2,934 (1.2)	5·20 (3·17, 8·51)	<0·001	8·65 (5·19, 14·42)	<0·001

*Cox proportional hazards regression; **Adjusted for the competing risk of deaths from causes other than neurodegenerative disease

© 2021 Russell ER et al. *JAMA Neurology.*

eTable 3: Date of birth and risk of neurodegenerative disease

	Former Soccer Players	Matched Population Controls	Hazard Ratio (95% CI)	*P**	Subhazard ratio (95% CI)	*P***
	**n/total n (%)**	**n/total n (%)**				
**1910-1929**	61/181 (33.7)	53/543 (9.8)	3·78 (2·26, 6·35)	<0·001	5·49 (3·11, 9·68)	<0·001
**1930-1949**	219/1,559 (14.0)	188/4,677 (4.0)	3·66 (2·85, 4·69)	<0·001	4·14 (3·29, 5·22)	<0·001
**1950-1969**	34/3,402 (1.0)	22/10,206 (0.2)	5·11 (2·77, 9·43)	<0·001	6·22 (3·41, 11·37)	<0·001

*Cox proportional hazards regression; **Adjusted for the competing risk of deaths from causes other than neurodegenerative disease

© 2021 Russell ER et al. *JAMA Neurology.*
